# Blood biomarker fingerprints in a cohort of patients with *CHRNE-*related congenital myasthenic syndrome

**DOI:** 10.1186/s40478-025-01946-9

**Published:** 2025-02-13

**Authors:** Adela Della Marina, Andrie Koutsoulidou, Daniel Natera-de Benito, Lars-Oliver Tykocinski, Marios Tomazou, Kristia Georgiou, Andreas Laner, Heike Kölbel, Andres Nascimento, Carlos Ortez, Angela Abicht, Basant Kumar Thakur, Hanns Lochmüller, Leonidas A. Phylactou, Tobias Ruck, Ulrike Schara-Schmidt, Dipali Kale, Andreas Hentschel, Andreas Roos

**Affiliations:** 1 Department of Pediatric Neurology, Center for Neuromuscular Disorders in Children and Adolescents, University Hospital Essen, University Duisburg-Essen, Essen, Germany; 2 Department of Molecular Genetics, Function and Therapy, The Cyprus Institute of Neurology and Genetics, Nicosia, Cyprus; 3 Neuromuscular Unit, Department of Neurology, Sant Joan de Deu Hospital, Barcelona, Spain; 4 Applied Research in Neuromuscular Diseases, Institut de Recerca Sant Joan de Déu, Barcelona, Spain; 5 Center for Biomedical Research Network on Rare Diseases (CIBERER), ISCIII, Barcelona, Spain; 6 Department of Hematology, Oncology and Rheumatology, Internal Medicine V, Medical Faculty, University Hospital Heidelberg, Heidelberg, Germany; 7 Department of Bioinformatics, The Cyprus Institute of Neurology and Genetics, Nicosia, Cyprus; 8 Medical Genetics Center, Munich, Germany; 9 Friedrich-Baur Institute, Ludwig Maximilian University, Munich, Germany; 10 Cancer Exosome Research Lab, Department of Pediatric Hematology and Oncology, University Hospital Essen, Essen, Germany; 11 Children’s Hospital of Eastern Ontario Research Institute, Ottawa, Canada; 12 Division of Neurology, Department of Medicine, The Ottawa Hospital, Ottawa, Canada; 13 Brain and Mind Research Institute, University of Ottawa, Ottawa, Canada; 14 Centro Nacional de Análisis Genómico (CNAG), Barcelona, Spain; 15 Department of Neurology, Medical Faculty, University Hospital Düsseldorf, Heinrich-Heine- University Düsseldorf, Düsseldorf, Germany; 16 Department of Neurology, BG & Heimer Institute for Muscle Research, University-Hospital Bergmannsheil Bochum, Ruhr-University, Bochum, Germany; 17 Leibniz-Institut für Analytische Wissenschaften -ISAS- e.V, Dortmund, Germany

**Keywords:** CMS biomarkers, CMS extracellular vesicles, CMS white blood cells, CMS miRNA, CMS metabolites

## Abstract

Mutations in *CHRNE* encoding the epsilon subunit of acetylcholine receptor result in impaired neuromuscular transmission and congenital myasthenic syndrome (CMS) with variying severity of symptoms. Although the pathophysiology is well-known, blood biomarker signatures enabling a patient-stratification are lacking. This retrospective two-center-study includes 19 recessive *CHRNE*-patients (AChR deficiency; mean age 14.8 years) from 13 families which were clinically characterized according to disease severity. 15 patients were classified as mildly and 4 patients as moderate to severely affected. Seven known pathogenic and one unreported variant (c.1032 + 2_1032 + 3delinsGT) were identified. Biomarker discovery was carried out on blood samples: proteomics was performed on white blood cells (WBC; *n* = 12) and on extracellular vesicles (EV) purified from serum samples (*n* = 7) in addition to amino acid profiling (*n* = 9) and miRNA screening (*n* = 18). For miRNA studies, 7 patients with other CMS-subtypes were moreover included. WBC-proteomics unveiled a significant increase of 7 and a decrease of 36 proteins. In silico studies of these proteins indicated affection of secretory granules and the extracellular space. Comparison across patients unveiled increase of two vesicular transport proteins (SCAMP2 and SNX2) in severely affected patients and indeed EV-proteomics revealed increase of 7 and decrease of 13 proteins. Three of these proteins (TARSH, ATRN & PLEC) are known to be important for synaptogenesis and synaptic function. Metabolomics showed decrease of seven amino acids/ amino acid metabolites (aspartic and glutamic acids, phosphoserine, amino adipate, citrulline, ornithine, and 1-methyhistidine). miRNA-profiling showed increase miR − 483 − 3p, miR-365a-3p, miR − 365b − 3p and miR-99a, and decrease of miR-4433b-3p, miR-6873-3p, miR-182-5p and let-7b-5p in *CHRNE*-patients whereas a comparison with other CMS subtypes showed increase of miR − 205 − 5p, miR − 10b − 5p, miR-125a-5p, miR-499-5p, miR-3120-5p and miR − 483 − 5p and decrease of miR − 1290. Our combined data introduce a molecular fingerprint on protein, metabolic and miRNA level with some of those playing different roles along the neuromuscular axis.

**Supplementary Information:**

The online version contains supplementary material available at 10.1186/s40478-025-01946-9.

## Introduction

The identification of congenital myasthenic syndrome (CMS)-associated mutations in genes specifically expressed at the neuromuscular junction (NMJ) contributed to the discovery of molecules involved in the NMJ signal transduction. Hereby, major CMS-causes are pathogenic variants affecting genes encoding the Acetyl-Choline-Receptor (AChR) subunits, namely, the α1 subunit (*CHRNA1*), β1 subunit (*CHRNB1*), δ subunit (*CHRND*), and ε subunit (*CHRNE*). Moreover, concomitant characterization of the mutant molecules enabled identification of underlying physiological mechanisms and strengthened the understanding of how synaptic signaling molecules mediate AChR clustering and NMJ functioning. Secreted molecules facilitating proper communication between the nerve and the muscle are here of significant importance. These molecules include agrin, ACh, Wnt, neuregulin-1, Ctgf, Rspo2, and Fgf18 [1].

Disrupted neuromuscular transmission results in muscular fatigability and weakness as a predominant clinical symptom. Pathogenic variants affecting genes encoding for proteins associated with post-synaptic defects are accounted for 87% of diagnosed cases [2, 3]. Among these, the gene most frequently harboring pathogenic variants is *CHRNE* (MIM# 100725), encoding the epsilon subunit of Acetyl Choline Receptor (AChR). These variants result in loss-of-function and deficiency of AChR at the endplate [2]. Here, NM_000080.4:c.1327delG(p.Glu443LysfsTer64), originating from the Roman population and NM_000080.4:c.1353dupG(p.Asn452fs) originating from the Maghreb, Spain and Portugal, are particularly frequent as bi-allelic pathogenic variant in Europe [4, 5].

*CHRNE*-patients are a clinically distinct group, with all patients presenting with ptosis and ophthalmoparesis. Onset of clinical symptoms occurs in the most within the first two years of life, rarely onset in the adulthood have been reported [6]. Clinical presentation and severity may vary enormously from mildly affected patients with isolated ocular symptoms (ophthalmoplegia and ptosis) to moderate or severe generalized muscle weakness. The symptoms usually stabilize in adulthood, but in some cases with moderate to severe muscular weakness with reduced walking distance or loss of the ambulation may persist [7–10]. Variability in clinical manifestation even in families carrying the same pathogenic variant in *CHRNE* have already been reported [8–10]. However, there is also indication of genotype-phenotype correlations: the clinical manifestation related to the c.1353dupG variant identified in 14 families of North African origin was relatively homogeneous with all patients showing moderate hypotonia and oculobulbar involvement, mild and stable disease course, and good response to cholinesterase inhibitors [5].

Although the genetic landscape of *CHRNE*-related CMS is well-studied, the knowledge of biomarkers – especially such of pathophysiological relevance – is still limited. In particular, there is a persisting need for minimal-invasive biomarkers which may enable a robust patient stratification. As the CHRNE protein has been identified in white blood cells (WBC) [11], WBC might be suitable for the discovery and monitoring of minimal-invasive but cellular biomarker fingerprints of pathophysiological relevance.

Here, we report on a cohort of 19 patients from 13 different families (Tables 1 and 2) harboring pathogenic *CHRNE* variants (8 different genotypes), including three discordant families presenting varying intra-familial severity of symptoms, in combination with protein data obtained from WBC (*n* = 12) and extracellular vesicles (EV) purified from serum samples (*n* = 7), as well as metabolic data and miRNA data from serum samples (*n* = 9 and 18, respectively) (Table 1 & Supplementary Table 1 as well as Supplementary Fig. 1). Proteomic signature of WBC and proteomics on EVs resulted in the identification of dysregulated proteins playing known roles along the neuromuscular axis. Metabolic profiling in sera enabled the definition of a decrease of seven amino acid/ amino acid metabolites in terms of a metabolic fingerprint in *CHRNE*-related CMS patients. Of note, several of these metabolites are also known to play essential roles in synaptic functions. The miRNA sequencing studies identified six miRNAs characteristic for *CHRNE*-related CMS, several of which are known to be important for proper muscle function and/or neuromuscular transmission.

**Table 1:** Overview of demographic and clinical severity scoring as well as applied therapeutic intervention approaches of*CHRNE*-patients included in the study. m = months, me = meters, na = not applicable, nd = not determinated, PS = pyridostigmine, DAP = diaminopyridine, eph = ephedrine, Sal = Salbutamol

Patient	Family	Sex	Descent	Age	Age symptom onset	Prenatal symptoms	Delayed motor milestones	Opthalmoplegia	Ptosis	Diplopia	Hypomimia	Bulbar symptoms	Proximal weakness	Distal weakness	Neck muscle weakness	Scoliosis	Reduced walking distance	Wheelchair	Progressive course	Fluctuating course	Worsening during infection	Age start therapy	Walking distance	Therapy	Severity	Analytical approaches
1	1	f	Turkey	13	0	-	-	+++	++	-	+	+	++	+	+	-	+	-	-	+	+	4 m	800me	PS, DAP	2	WBC, miRNA, EV, Met
2	1	f	Turkey	19	4 m	-	-	++	++	+	+	+	++	+	+	-	-	-	+	+	+	12 m	normal	PS, DAP	2	WBC, miRNA, EV, Met
3	1	f	Turkey	28	0	-	+	+++	+++	-	+	-	+++	++	++	+	++	-	+	+	+	nd	500me	PS, DAP	3	WBC, miRNA
4	2	f	Syria	4	3 m	-	-	++	+	-	-	-	-	-	-	-	-	-	-	-	-	8 m	normal	none	1	WBC, miRNA, Met
5	2	m	Syria	14	0	-	+	+++	+++		++	+	+++	+	++	-	+++	+	+	-	+	9y	70me	PS, 3,4 DAP, Eph	4	WBC, miRNA, EV
6	2	m	Syria	17	infant	-	-	++	++	-	-	-	+	-	-	-	-	-	-	-	+	nd	normal	PS, stopped	1	WBC, miRNA, EV, Met
7	3	f	Turkey	5	9 m	-	-	++	++	-	+	++	++	++	++	-	++	-	+	+		2y	1 km	PS, DAP	2	WBC, miRNA
8	3	f	Turkey	18	5y	-	-	++	++	-	+	++	+++	++	++	+	+++	+	+	-	-	16y	100me	PS, DAP	4	WBC, miRNA, EV, Met
9	4	f	Turkey	13	infant	-	-	+	+	-	-	-	++	-	+	-	+	-	+	-		12y	normal	PS	2	WBC, Met
10	5	f	Serbia	15	0	nd	+	+++	++		+	++	++	+	+	-	++	-	+	+	+	1y	500me	PS, Sal	3	WBC, miRNA, Met
11	6	m	Afghanistan	13	0	-	-	++	++	-	+	-	+	-	-		-	-	-	-	-	9y	normal	PS	2	WBC, miRNA, EV, Met
12	7	m	Croatia/Italy	16	0	-	+	++	++	+	+	+	++	+	-	-	+	-	-	+	+	3y	normal	PS, DAP, Sal	2	WBC, miRNA, EV, Met
13	8	m	Marocco	1	0	-	-	+++	++	na	+	-	-	-	-	-	na	na	-	-	+	1y	na (age < 2y)	PS	1	miRNA
14	9	f	Marocco	7	0	-	+	++	++	-	+	-	-	-	-	-	-	-	-	-	+	2y	normal	PS	1	miRNA
15	10	m	Spanish (Roma)	18	0	-	+	+++	++	+	+	+	++	+	+	-	+	-	-	-	+	4y	1 km	PS	2	miRNA
16	10	m	Spanish (Roma)	20	0	-	-	+++	++	-	+	-	-	-	-	-	-	-	-	-	+	4y	normal	PS	1	miRNA
17	11	f	Spanish	30	0	-	-	+++	++	-	+	-	-	-	-	-	-	-	-	-	+	4y	normal	PS	1	miRNA
18	12	f	Spanish	20	infant	-	-	+	++	-	+	+	+	+	+	+	+	-	-	+	+	3y	1 km	PS	2	miRNA
19	13	f	Bulgaria (Roma)	10	2y	nd	+	+++	++	-	+	+	++	-	+	-	+	-	+	-	+	5y	1 km	PS, Sal	2	miRNA

**Table 2:** Overview of genotypes of *CHRNE*-patients included in the study. hm = homozyhous; cht = compound heterozyhous

Patient	Family	Genetic variant (inheritance)	HGVS nomenclature	Classifiaction in ClinVar / other information
1	1	c.452_454del (p.Glu151del) (hm)	NM_000080.4(CHRNE): c.452_454del (p.Glu151del)	conflicting: VUS, likely path, path
2	1	c.452_454del (p.Glu151del) (hm)	NM_000080.4(CHRNE): c.452_454del (p.Glu151del)	conflicting: VUS, likely path, path
3	1	c.452_454del (p.Glu151del) (hm)	NM_000080.4(CHRNE): c.452_454del (p.Glu151del)	conflicting: VUS, likely path, path
4	2	c.1032 + 2_1032 + 3delinsGT (hm)	NM_000080.4(CHRNE): c.1032 + 2_1032 + 3delinsGT, r.spl?, p.(Val345ArgfsTer52)	SpliceAI: donor loss and donor gain 1 nucleotide upstream, out-of-frame effect
5	2	c.1032 + 2_1032 + 3delinsGT (hm)	NM_000080.4(CHRNE): c.1032 + 2_1032 + 3delinsGT, r.spl?, p.(Val345ArgfsTer52)	SpliceAI: donor loss and donor gain 1 nucleotide upstream, out-of-frame effect
6	2	c.1032 + 2_1032 + 3delinsGT (hm)	NM_000080.4(CHRNE): c.1032 + 2_1032 + 3delinsGT, r.spl?, p.(Val345ArgfsTer52)	SpliceAI: donor loss and donor gain 1 nucleotide upstream, out-of-frame effect
7	3	c.1327del (p.Glu443LysfsTer64) (hm)	NM_000080.4(CHRNE): c.1327del (p.Glu443LysfsTer64)	pathogenic
8	3	c.1327del (p.Glu443LysfsTer64) (hm)	NM_000080.4(CHRNE): c.1327del (p.Glu443LysfsTer64)	pathogenic
9	4	c.1327del (p.Glu443LysfsTer64) (hm)	NM_000080.4(CHRNE): c.1327del (p.Glu443LysfsTer64)	pathogenic
10	5	c.1327del (p.Glu443LysfsTer64) (hm)	NM_000080.4(CHRNE): c.1327del (p.Glu443LysfsTer64)	pathogenic
11	6	c.1327del (p.Glu443LysfsTer64) (hm)	NM_000080.4(CHRNE): c.1327del (p.Glu443LysfsTer64)	pathogenic
12	7	c.1291G > C p.Ala421Pro (cht)	NM_000080.4(CHRNE): c.1291G > C (p.Ala431Pro)	pathogenic
		c.1441 C > T, p.Arg481Ter (cht)	NM_000080.4(CHRNE): c.1441 C > T (p.Arg481Ter)	pathogenic
13	8	c.1373_1375del (p.Cys458del) (hm)	NM_000080.3(CHRNE): c.1373_1375del (p.Cys458del)	pathogenic
14	9	c.1353dup (p.Asn452fs) (hm)	NM_000080.4(CHRNE): c.1353dup (p.Asn452fs)	pathogenic
15	10	c.1327del (p.Glu443LysfsTer64) (hm)	NM_000080.4(CHRNE): c.1327del (p.Glu443LysfsTer64)	pathogenic
16	10	c.1327del (p.Glu443LysfsTer64) (hm)	NM_000080.4(CHRNE): c.1327del (p.Glu443LysfsTer64)	pathogenic
17	11	NM_000080.4(CHRNE): c.130dup (p.Glu44fs) (cht)	NM_000080.4(CHRNE): c.130dup (p.Glu44fs)	pathogenic
		c.1353dup (p.Asn452GlufsTer4) (cht)	NM_000080.4(CHRNE): c.1353dup (p.Asn452GlufsTer4)	pathogenic
18	12	c.127_128insG; p.Glu44fs (hm)	NM_000080.4(CHRNE): c.130dup (p.Glu44GlyfsTer3)	pathogenic
19	13	c.1327del; p.Glu443Lysfs*64 (hm)	NM_000080.4(CHRNE): c.1327del (p.Glu443LysfsTer64)	pathogenic

Thus, our combined biomarker screening data hinted toward pathophysiologies underlying in recessive *CHRNE*-related CMS (AChR deficiency) and enabled us to obtain novel insights into this rare disease in terms of (i) a broader understanding of the etiopathology on different molecular layers and (ii) the first introduction of minimal-invasive biomarkers including cellular and versicular marker proteins as well as circulating metabolites and miRNAs. Hence, we here introduce the first molecular fingerprint holding the potential to serve as a minimal-invasive biomarker of pathophysiological relevance in *CHRNE*-related CMS.

## Materials and methods

### CMS patients

In this study, 19 recessive *CHRNE*-patients (from 13 different families; age range from 1 to 30 years, mean 14.75 years (Table 1) were clinically investigated and treated in two European centers: the neuromuscular centre of the department of pediatric neurology of Duisburg-Essen University (University Hospital Essen, Germany) (*n* = 12 patients) as well as 7 patients from Hospital Sant Joan de Déu, Barcelona, Spain. For all patients, molecular genetic data (obtained in terms of a routine diagnostic work-up of these cases) were available and are listed along with demographic and clinical findings in Table 2. All patients gave informed constent for the genetic studies and biomarker sampling. In the light of our main inclusion criterion of recessive *CHRNE*-based CMS, only one of 19 patients (patient 12) harbours a compound heterozygous variant (p.Ala431Pro) which has been linked to subtle AChR kinetic defect in terms of a fast channel syndrome [12].

For proteomics-based monitoring of protein signatures in white blood cells, 3.7 ml EDTA blood was collected from 12 patients. For purification of EVs, amino acid profiling and miRNA screening 500 µl serum were used from each patient. Because of numeric variation of availability of patient blood samples for certain methods and subgroups, numbers of patient-derived samples utilized per applied analytical approach varies as follows: miRNA studies, *n* =18; global proteomics on white blood cells, *n* = 12; proteomic analysis of extracellular vesicles isolated from serum, *n* = 7 & metabolic profiling of serum, *n* = 9. A further overview of applied analytical approaches on the individual patient level is provided in Table 1. All biosamples were cryopreserved at − 80 °C prior to analyses. Supplementary Fig. 1 provides an overview of the different sample numbers (*CHRNE*-patients, normal disease controls (NDC) & further CMS genotypes (CMS controls serving as disease controls) included in the different studies. Our study was approved by the ethical committee of the University Hospital Duisburg-Essen (19-9011-BO) and for Spanish patients by ethical comitee of the Institut de Recerca Sant Joan de Deu (PIC-147-23).

Mean age of none-disease controls used in this study was 19.6 years with a nine male and 8 female participants.

The study was conducted in accordance with the principles of the Declaration of Helsinki. The data that support the findings of this study are available from the corresponding author upon request.

### Clinical stratification

The clinical presentation was stratified based on the clinical examination conducted by three senior clinicians (US-S, ADM, and DNdB) with a long-term experience in the care of CMS patients as followed: only ocular symptoms (opthalmoplegia and ptosis): severity class 1; combination of ocular symptoms and mild generalized weakness (walking distance reduced): severity class 2; combination of ocular symptoms and moderate generalized weakness: severity class 3; combination of ocular symptoms and severe muscular weakness (wheelchair bound): severity class 4. Clinical stratification per patient is detailed in Table 1. Due to partly very young age of patients and lack of cooperation, no standartised score for (autoimmune) myasthenia was used.

### Proteomic profiling on *CHRNE*-patient derived white blood cells (WBC)

After isolation and purification, white blood cells were stored at -80 °C until further processing for proteomic profiling. Isolation of WBC, protein extraction, determination of protein concentration and further samples processing for liquid-chromatography coupled to tandem mass spectrometry in a data-independent manner were carried out as described previously [13].

### EV isolation from *CHRNE*-patient derived serum and proteomic profiling

After collection of serum derived from *CHRNE*-patients, 500 µl aliquots were used for the isolation and purification of EVs: EVs were purified from serum by size exclusion chromatography (SEC) using 35 nm qEVoriginal Gen 2 columns (Izon Science Ltd.) followed by ultra-filtration according to the manufacturer´s instructions. Briefly, 500 µl serum were layered on a SEC column and allowed to run into the column, followed by 2 ml freshly filtered (0.22 μm) phosphate buffered saline (PBS). Next, 2.5 ml filtered PBS was added to the columns, the flow-through was collected and filtered directly (Amicon Ultra-4, 100 kDa, Merck), thereby reducing the volume to around 300 µl. The total number and size of the EVs was determined by nanoparticle tracking analysis (ZetaView PMX-220, Particle Metrix (settings: Sensitivity 80, Shutter 100, Maximum Particle Size 1000, Minimum particle size 10, Maximum brightness 255, Minimum brightness 30)), the EV protein concentration by BCA assay (Micro BCA Protein Assay Kit, Thermo Fisher Scientific). For the study of EVs, in addition to 7 *CHRNE*-patients, samples from two *CHAT*-, one *CHRNA1*- and one *SLC18A3*-patient [14] were included as disease controls (Supplementary Table 1).

Next, snap-frozen EVs were processed for proteomic profiling as follow: proteins were precipitated by adding ice cold acetone in a 3 fold excess and storing the samples overnight at -20 °C.

After centrifugation at 4 °C for 20 min at 20.000 g, acetone was removed and the samples were allowed to dry under a flow hood. The dried protein pellets were solubilized by adding 200 µl of 50 mM Tris-HCl buffer (pH 7.8), 5% SDS and cOmplete ULTRA protease inhibitor (Roche). Sample preparation for mass spectrometry was carried out as described previously [13].

Mass spectrometry analysis was conducted using a timsTOF HT instrument (Bruker Daltonics, Germany) operating in data-dependent acquisition (DDA) Parallel Accumulation–Serial Fragmentation (PASEF) mode. Each acquisition cycle comprised one MS1 scan followed by ten PASEF MS/MS scans. Ion accumulation and ramp times were configured to 100 ms. The analysis encompassed ions within an ion mobility range (1/K0) of 1.6 Vs cm − 2 to 0.6 Vs cm − 2 and an MS scan range of 100–1700 m/z. Collision energy was dynamically adjusted based on ion mobility, starting at 59 eV for 1/K0 = 1.6 VS cm − 2 and decreasing to 20 eV for 1/K0 = 0.6 VS cm − 2. A dynamic exclusion period of 0.4 min was applied.

The timsTOF data were analyzed using PeaksOnline software (Bioinformatics Solutions Inc.). The database search parameters were configured as follows: precursor mass tolerance was set to 15 ppm and fragment mass tolerance to 0.1 Da. Trypsin was used as the enzyme, with up to two missed cleavages allowed. For protein identification, a human database downloaded from UniProt (January 2023) was used. Carbamidomethylation was set as a fixed modification, and oxidation of methionine as a variable modification. The false discovery rate (FDR) was set to 1% for peptide-spectrum matches (PSMs), peptides, and proteins.

The mass spectrometry proteomics data have been deposited to the ProteomeXchange Consortium via the PRIDE [15] partner repository with the dataset identifier PXD054347.

### Metabolic analyses

Metabolites were extracted from sera and derivatized as previously reported [16], with few modifications. In brief, 50 µL of human serum was transferred into a 1.5 mL tube and 200 µL of an ice-cold 1:1 mixture of ACN and methanol containing an internal standard (ISTD, D5-Tryptophan, Cambridge Isotope Lab, 25 µM) was added. Next, samples were vortexed briefly. An overight incubation (16 h, -80˚C) was subsequently performed for protein precipitation. Next day, samples were centrifuged at 15,000 × g for 10 min. The supernatant was transferred to a new 1.5 mL tube. Next, 25 µL of the supernatant was derivatized directly as described later. A pooled serum quality control (PQC) sample was prepared by mixing 10 µL of each sample and was accordingly used to monitor the performance of the analytical instruments. Calibration curves for selected amino acid standards were prepared in the range 2.5 to 2500 µmoles/L (except for cystine: 1.25 to 1250 µmoles/L) from the amino acid standard (Sigma Aldrich AAS18).

For derivatization, 2.85 mg of AQC was dissolved in 1 mL anhydrous ACN. Next, 60 µL of borate buffer (pH 8.8) was added to each sample and samples were vortexed. Next, 20 µL of AQC solution was added and samples were vortexed and centrifuged (1 min at 15,000 × g). Afterwards, samples were kept for 1 min at RT. This was followed by a 10 min incubation step at 55 ˚C. The samples were then vortexed and centrifuged at 15,000 × g for 5 min. Finally, 1 µL of sample was injected on LC-MS. AQC derivatized serum extracts samples were acquired at random order. Precisely, AQC derivatized serum extracts were separated on an Vanquish Duo LC-system using an Acclaim Vanquish C18, (2.1 mm ×150 mm, 2.2 µM) column. Elution was carried out with a binary gradient of mobile phase A (50 mM Ammonium formate buffer (pH 2.9)); and mobile phase B (100% ACN). The gradient was as follows: 0–0.4 min 0.5%B; 0.4–3.9 min 0.5–5.2%B; 3.9–5.9 min 5.2–9.2%B; 5.9–6.3 min 9.2–14.0%B; 6.3–8.2 min 14.0-19.2%B;8.2–10 min 19.2–19.5% B; 10–10.05 min 19.5–90% B; 10.05–10.5 min 90% B; 10.5–10.55 min 90 − 0.5% B; and 10.55–16.0 min 0.5% B. The column was maintained at 45 ˚C, and flow rate was 500 µL/min. The samples were analyzed by Exploris 240 instrument in positive ionization mode using full scan + PRM scan mode using following instrument settings: Spray voltage 3.5 kV, Sheath gas 35 (arb), Aux gas 10 (arb), Sweep gas 0 (arb), Capillary temperature 300 ˚C, Vaporizer temp 310 ˚C, Full scan mass range 200–600 mz, Full scan resolution 60,000, PRM resolution 15,000; HCD Collision energy stepped 15,20,25. Full scan MS Data was processed using Skyline software. The characteristic fragment ion 171.0155 originated from the AQC molecule was used as a qualifier ion for all amine containing metabolites. The following 17 proteinogenic amino acids were identified, and quantified based on external calibration curves of standard: L-Alanine (Ala), L-Arginine (Arg), L-Aspartic Acid (Asp), L-Glutamic Acid (Glu), Glycine (Gly), L-Histidine (His), L-Leucine (Leu), L-Lysine (Lys), L-Methionine (Met), L-Phenylalanine (Phe), L-Proline (Pro), L-Serine (Ser), L-Threonine (Thr), L-Tyrosine (Tyr), L-Valine (Val), L-Isoleucine (Ile), and L-Cystine (Cys). Quantitation of these amino acids was conducted by constructing an external standard curve as described previously.

Other amine containing metabolites namely Tryptophan (Trp), Phosphoserine (pSer), Taurine, Asparagine (Asn), Glutamine (Gln), Amino Adipate, Citrulline, β-Aminobutyric acid, Ornithine, 1-methylhistidine (1-MH), 3-methylhistidine (3-MH), and hydroxyproline (OH-Pro) were characterized based on MS/MS matching. For quantitative determination of these metabolites, D5-Tryptophan was used as an internal standard. To address statistical relevance, t-test comparison between all groups was conducted. The amino acids of those with p value ≥ 0.05 were removed from further comparison.

### Small RNA isolation and next generation sequencing

Total RNA, including miRNAs, was extracted from serum samples of 18 *CHRNE* patients, 7 disease control patients (1 *CHRNE*-related “slow-channel”, 1 *CHRNB1*, 1 *CHAT*, 2 *GFPT1*, 1 *CHRNA1* and 1 *SLC18A3*) and 12 healthy controls using the mirVana PARIS Kit (Applied Biosystems), following the manufacturer’s instructions (Supplementary Table 1). Libraries were prepared from 5 µl of RNA using the QIAseq miRNA Library Kit (Qiagen) according to the manufacturers’ instructions. Library concentrations were measured using Qubit™ dsDNA HS Assay Kit (Thermo Fisher). Quality and concentration of libraries were determined using an Agilent 2200TapeStation system with high sensitivity ScreenTape assay and reagents (Agilent Technologies) and by Real-Time PCR using the QIAseq Library Quant Assay Kit (Qiagen). Sequencing was performed on the Nextseq 2000-Illumina using the NextSeq 2000 P3 Reagents 100-cycle kit (Illumina).

### miRNA bioinformatic analyses

Fastq reads were pre-processed using the nextflow v23.10.1, nf-core/smrnaseq pipeline v2.3.0. Preprocessing included read quality control using FastQC v0.12.0, adapter removal, trimming and filtering using fastp v.0.23.4, contamination control using miRTrace v1.0.1 and read mapping using bowtie v1.3.1 against both the mature and hairpin reference sequences from miRbase (Release 22.1). The mapped reads counts were quantified using MIRTOP v0.4.25.

Differential Expressional (DE) analysis of miRNAs was performed using EdgeR (Bioconductor Release 3.17) in R v4.3. The following pairwise comparisons were performed for the *CHRNE* group against the healthy control group, *CHRNE* group against the other CMS-subtype patients and all CMS patients against healthy control group (while controlling for age). For each pairwise comparison the resulting log2 Fold Change (log2FC) and adjusted *p. values* were recorded for downstream analysis. DE of miRNAs was visualized using the ggplot2 v3.1 and EnhancedVolcano v1.15 packages in R.

The miRNAs with |log2FC| > 0.5 and adjusted *p. value* < 0.05 were selected for hierarchical clustering using the pheatmap package in R. In addition, validated gene targets for the selected miRNA from each set, were obtained using the multiMiR package in R (Bioconductor Release v3.17). Specifically, all known gene targets from mirecords, mirtarbase and tarbase, characterized as ‘validated’ through functional experiments were downloaded and filtered to remove genes identified from low confidence experiments such as a single degradome sequencing and HITS-CLIP labelled as ‘weak’ evidence.

The remaining gene sets were used for pathway enrichment using the clusterProfiler package in R (Bioconductor Release 3.17) along with org.Hs.eg.db for entrez, Ensembl and gene symbol mapping. The enrichment was performed using the OverRepresentation Analysis (ORA) functions of the package against the GO biological process (BP) molecular function (MF) and cellular component (CC) Gene Ontology (GO) databases as well as against the KEGG database. For each pairwise group comparison we performed a separate ORA analysis. The resulting pathways were visualized using the ggplot2 package in R.

## Results

### *CHRNE*-related phenotypes

19 patients (mean age 14.8 years, range 1.7–30 years) from 13 families were included (12 female and 7 males). In 18/19 patients, onset of first symptoms was neonatal or during the first months of life, only one patient (patient 8) developed her first symptoms at the age of five years. Whereas 15 patients were classified as mildly affected (group A: grade 1 and 2), 4 patients were classified as moderate or severely affected (group B: grade 3 and 4) (Table 1). All patients had moderate to severe ophthalmoparesis and fluctuating ptosis as a common clinical symptom. In 14/19 patients variable severity of proximal muscle weakness was present, while distal and/or neck muscle weakness were less common. Both, hypomimia and bulbar symptoms were present in 7 patients, further 6 presenting facial hypomimia without bulbar symptoms. Two patients had severe muscular weakness and needed wheelchair for longer distances. None of the patients required respiratory support. Intellectual diasability was present in 4/19. Two patients were therapy naive, both of whom were only treated temporarily with pyridostigmine and discontinued due to subjective lack of therapeutic benefit (family 2). However, 11 patients were on monotherapy with pyridostigmine (PS), 7 received combined therapy with 3,4 Diaminopyridine (DAP) and 4 with salbutamol/ephedrine (Table 1). Start of therapy varied from the age of 4 months to 16 years. None of the patients received another additional therapy.

### Molecular genetics findings

The most frequent pathogenic variant NM_000080.4:c.1327del (p.Glu443Lysfs*64) (aka: c.1267delG) was present in 8/19 patients (Table 2).

Seven known pathogenic variants in *CHRNE* were identified in addition to one variant previously not reported in literature (NM_000080.4:c.1032 + 2_1032 + 3delinsGT, r.spl?, p.?). Notably, hereby one family member was severely affected and presented with moderate to severe muscular weakness in combination with intellectual disability, whereas his two siblings were only mildly affected and showed predominant ocular symptoms. The variant which is absent from gnomAD v2.1.1 and affects the canonical splice donor site of exon 9. A bioinformatic prediction using SpliceAI suggests destruction of the physiological splice donor site and the generation of a new splice donor site a nucleotide 3’ of the physiological donor. This would lead to a frameshift with a subsequent stop codon (p.(Val345ArgfsTer52)). According to the American College of Medical Genetics and Genomics/ Association for Molecular Pathology (ACMG/AMP) classification guidelines [17] this variant can be classified as pathogenic (ACMG/AMP: PVS1, PM2_SUP, PP1).

Family 1 presents with a *CHRNE*-variant (NM_000080.4:c.452_454delAGG, p.(Glu151del)) and a phenotype compatible with *CHRNE*-related CMS including response to therapy with pyridostigmine and 3,4 DAP. This variant results in an in-frame deletion that is predicted to remove a single highly conserved amino acid from the extracellular domain of the encoded protein. Moreover, this variant is absent in gnomAD V2.1.1 and was detected in homozygous state in several unrelated families with *CHRNE*-associated phenotype and segregated with the disease in several families [18–21]. According to the ACMG/AMP classification guidelines this variant can be classified as likely pathogenic (ACMG/AMP: PS4_SUP, PM3, PM4, PP1, PM2_SUP).

**Fig. 1:**
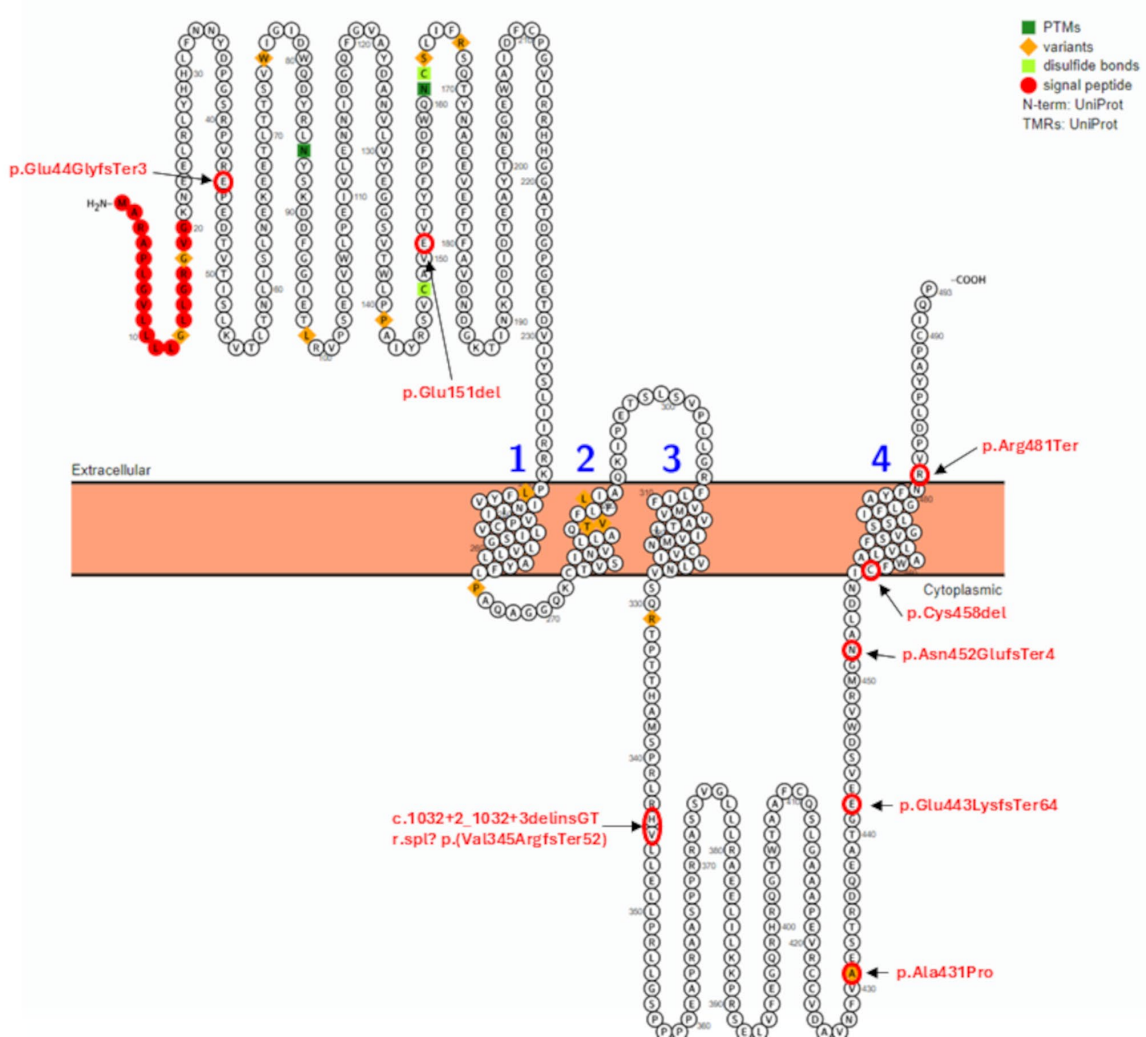
Schematic overview of distribution of *CHRNE*-variants identified in our patients across the protein structure. Protter: interactive protein feature visualization and integration with experimental proteomic data [46]

The other pathogenic variants along with results of their in silico prediction (where applicable) are listed in Table 2. An overview of the localization of all variants identified in our *CHRNE*-cohort is presented in Fig. 1.

### Proteomic profiling on white blood cells derived from *CHRNE*-patients

**Fig. 2:**
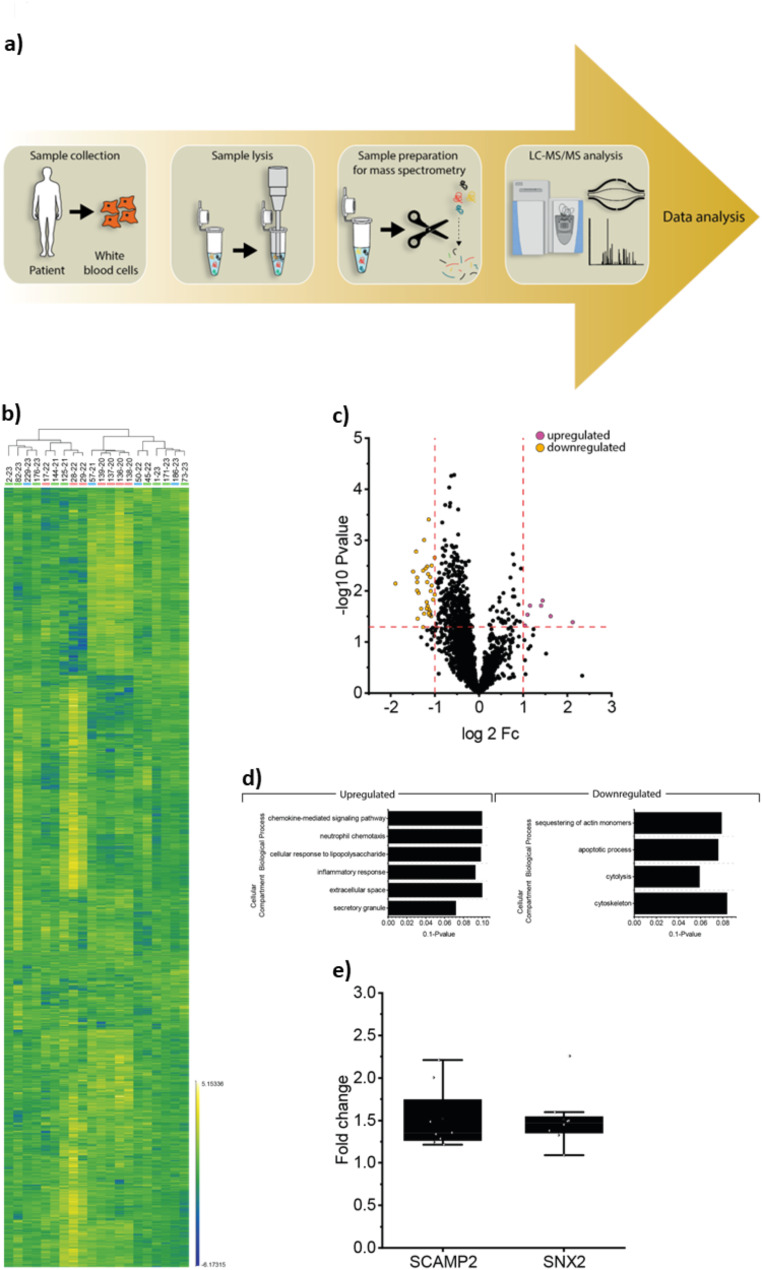
Study of protein signature of white blood cells derived from *CHRNE*-patients. (**a**) Schematic representation of proteomic workflow applied on white blood cells derived from *CHRNE*-patients and healthy controls. (**b**) Proteingenic changes are shown in the heatmap on the individual protein level. (**c**) Volcano plot depicting statistically significant dysregulated proteins in white blood cells of *CHRNE*-patients. Increased proteins are represented by purple dots wereas decreased proteins are represented by organge dots. (**d**) GO-term based in silico analysis of pathways affected by protein dysregulations affected by upregaulated and downregulated proteins, respectively. (**e**) Box plots highlighting the increase of SCAMP2 and SNX2 as two proteins relevant for vesicular transport in cells derived from *CHRNE*-patients compared to controls

Data-independent acquisition (DIA) is one of the most powerful and reproducible proteomic technologies for large-scale digital qualitative and quantitative research. The aim of this sub-study was to use DIA-based proteomic methodologies for the identification of minimal-invasive but cellular biomarkers that are differentially abundant in blood samples derived from *CHRNE* patients. Of note, CHRNE abundance was demonstrated in the framework of one of our previous studies [11] declaring white blood cells as a suitable model to identify minimal-invasive cellular markers. Doing so, DIA-based proteomics was applied on white blood cells derived from 12 *CHRNE*-patients (Fig. 2a & Supplementary Fig. 1) and a comparison of the proteomic signature of all patients versus controls unveiled a significant increase of 7 and a decrease of 36 proteins (Fig. 2b and c & Supplementary Table 2). GO-term based in silico studies of these dysregulated proteins showed that increased proteins impact on diverse biological processes including chemokine-mediated signaling pathway, neutrophil chemotaxis, cellular response to lipopolysaccharide and to inflammation (Fig. 2d). Cellular compartments affected by decreased proteins are mainly secretory granules and the extracellular space (Fig. 2d). Moreover, GO-term based in silico studies unraveled that the decreased proteins impact on apoptotic processes, cyotolysis and cytoskeleton (sequestering of actin monomers), and mitochondrial electron transport (cytochrome c to oxygen). When comparing proteomic signatures of *CHRNE*-patients with a mild phenotype (classified as grade 1 or 2; see Table 1) versus such with a moderate to severe phenotype (classified as grade 3 or 4; see Table 1), two proteins (SCAMP2 and SNX2; see Supplementary Table 2) were significantly increased distinguishing these two patient groups (Fig. 2e). In the global comparison of all *CHRNE*-patients versus normal controls SCAMP2 shows a mild but statitiscally not relevant elevation of 1.28-fold (*p*-Anova = 0.25). In the same comparison, SNX2 also shows a mild 1.20-fold elevation which is also statistically not significant (*p*-Anova = 0.24). However, SCAMP2 and SNX2 are both involved in vesicle transport hinting toward affection of extracellular exosomes as a significant determinant in the molecular etiology of *CHRNE*-related CMS.

### Purification and analysis of serum-derived EV

**Fig. 3:**
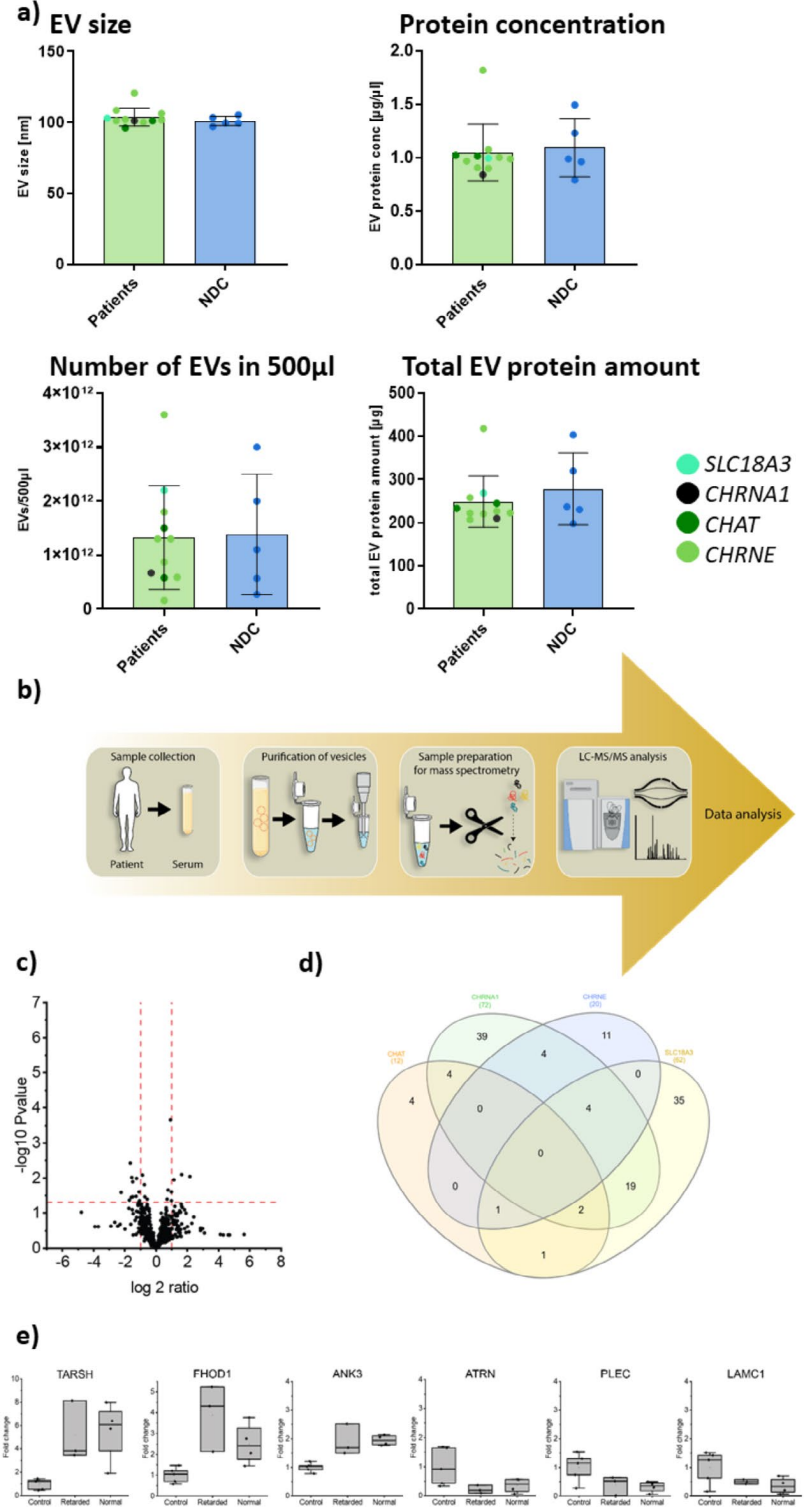
Study of EVs purified from blood derived of *CHRNE*-related CMS patients. (**a**) Characterization of EVs purified from sera derived from CMS-patients and age-matched controls including determination of size, protein concentration number per 500 µl and total protein amount. (**b**) Schematic representation of proteomic workflow applied on EVs derived from CMS patients and healthy controls. (**c**) Volcano plot depicting statistically significant dysregulated proteins in EVs of *CHRNE*-patients. Increased proteins are represented by purple dots wereas decreased proteins are represented by organge dots. (**d**) Venn diagram showing overlaps of dysregulated EV proteins across the different CMS subtypes. (**e**) Box plots depicting TARSH, ATRN and PLEC as three proteins of major interest in addition to ANK3, LAMC1 and FHOD1 dysregulated in EVs of *CHRNE*-patients. No changes of these proteins were noted between patients with and without intellectual disability

Prompted by the results obtained on *CHRNE*-patient derived white blood cells, next we aimed to investigate the quality of EVs purified from serum. Here, quality control of purified EV revealed no differences insize, total number of EVs and overall protein concentration of EVs, between CMS patients (*CHRNE*-, *CHRNA1*-, *CHRNB1*,* CHAT*-, and *SLC18A3*-related) and controls (Fig. 3a). However, label-free mass spectrometric analyses of protein extracts of EVs derived from CMS-patients (seven *CHRNE*-patients and four disease controls: two *CHAT* patients, one *CHRNB1* and one *SLC18A3* patient respectively) and healthy controls (Fig. 3b) showed a significant dysregulation of 20 proteins in *CHRNE*-patients whereby 7 are increased and 13 decreased (Fig. 3c & Supplementary Table 3). A comparison of the different CMS genes revealed no overlap of dysregulated EV proteins across all subtypes. However, five EV proteins overlap between *CHRNE* and *SLC18A3* (FHOD1, ANK3, IGHG3, TFPI1 and HV70D) as well as four between *CHRNE* and *CHRNA1* (FHOD1, TARSH, ANK3 and IGHG3) (Fig. 3d). The EV proteins significantly dysregulated in *CHRNE*-patiens also included neurological relevant proteins: Target of Nesh-SH3 (TARSH; 4.48-fold increased) as well as Attractin (ATRN; decreased to 0.36 rest protein level) and Plectin (PLEC; decreased to 0.35 rest protein level) (Fig. 3e). These three proteins are known to be important for myelination, synaptogenesis and synaptic function [22–24]. In patients with intellectual diasability no differences in the level of this proteins was observed.

### Metabolic profiling on sera derived from *CHRNE*-patients

**Fig. 4:**
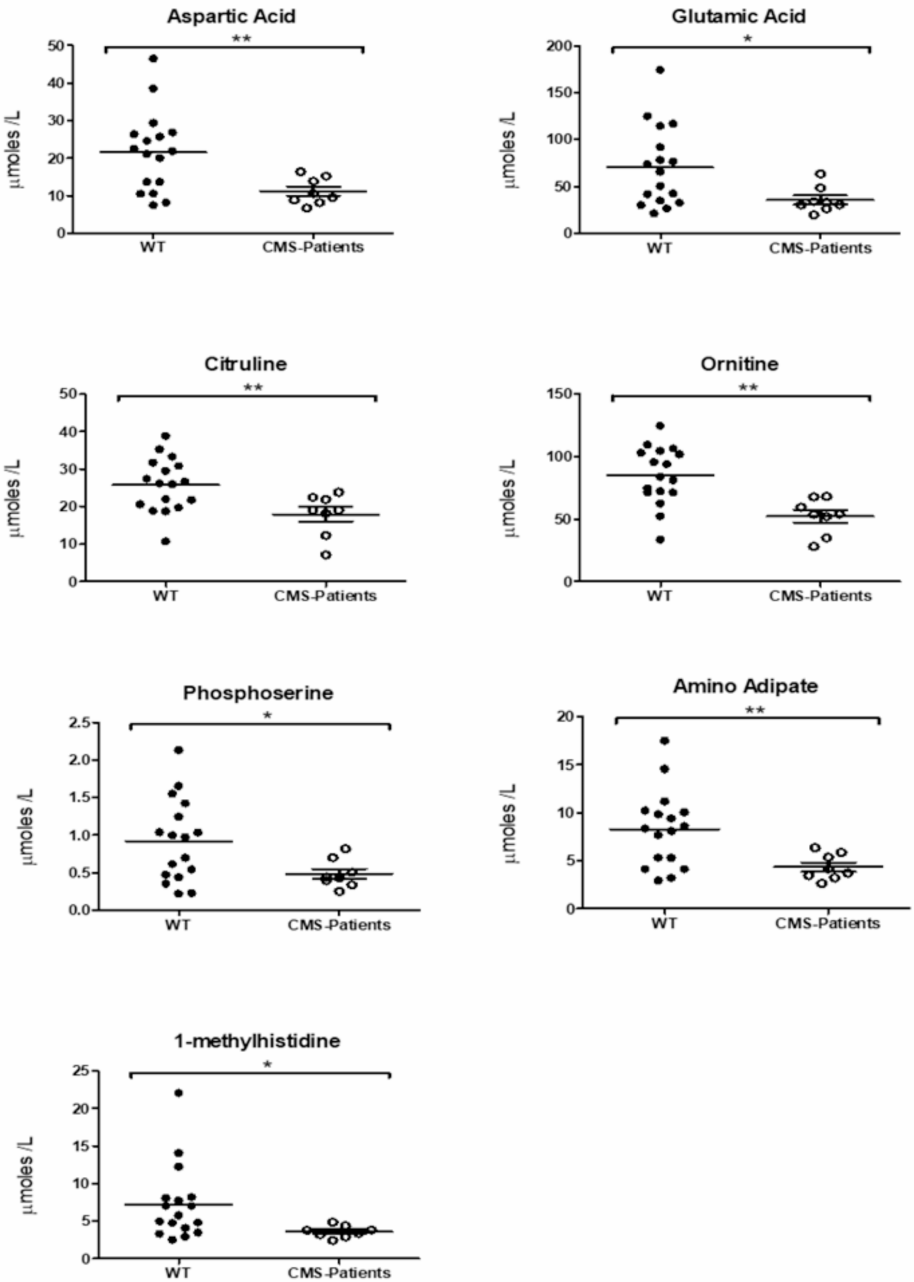
Study of metabolic status in sera derived from *CHRNE*-patients. Mass spectrometry unveils statistically significant decrease of aspartic and glutamic acids, phosphoserine, amino adipate, citrulline, ornithine & 1-methyhistidine

Taking the results of our unbiased proteomic profiling data on WBC indicative for mitochondrial vulnerability and thus metabolic activities based on decreased proteins into consideration, metabolic profiling was carried out on sera derived from 9 *CHRNE* patients, compared to 17 non-disease controls. This analytical approach unveiled a significant decrease in seven amino acids/ amino acid metabolites: aspartic and glutamic acids, phosphoserine, amino adipate, citrulline, ornithine & 1-methyhistidine (Fig. 4; Supplementary Table 4), all involved in amino acid consumption and turn-over. Of note, no differences in the abundance of these seven metabolites were observed when comparing the different disease severity groups (data not shown).

### miRNA profiling on sera derived from *CHRNE*-patients

**Fig. 5:**
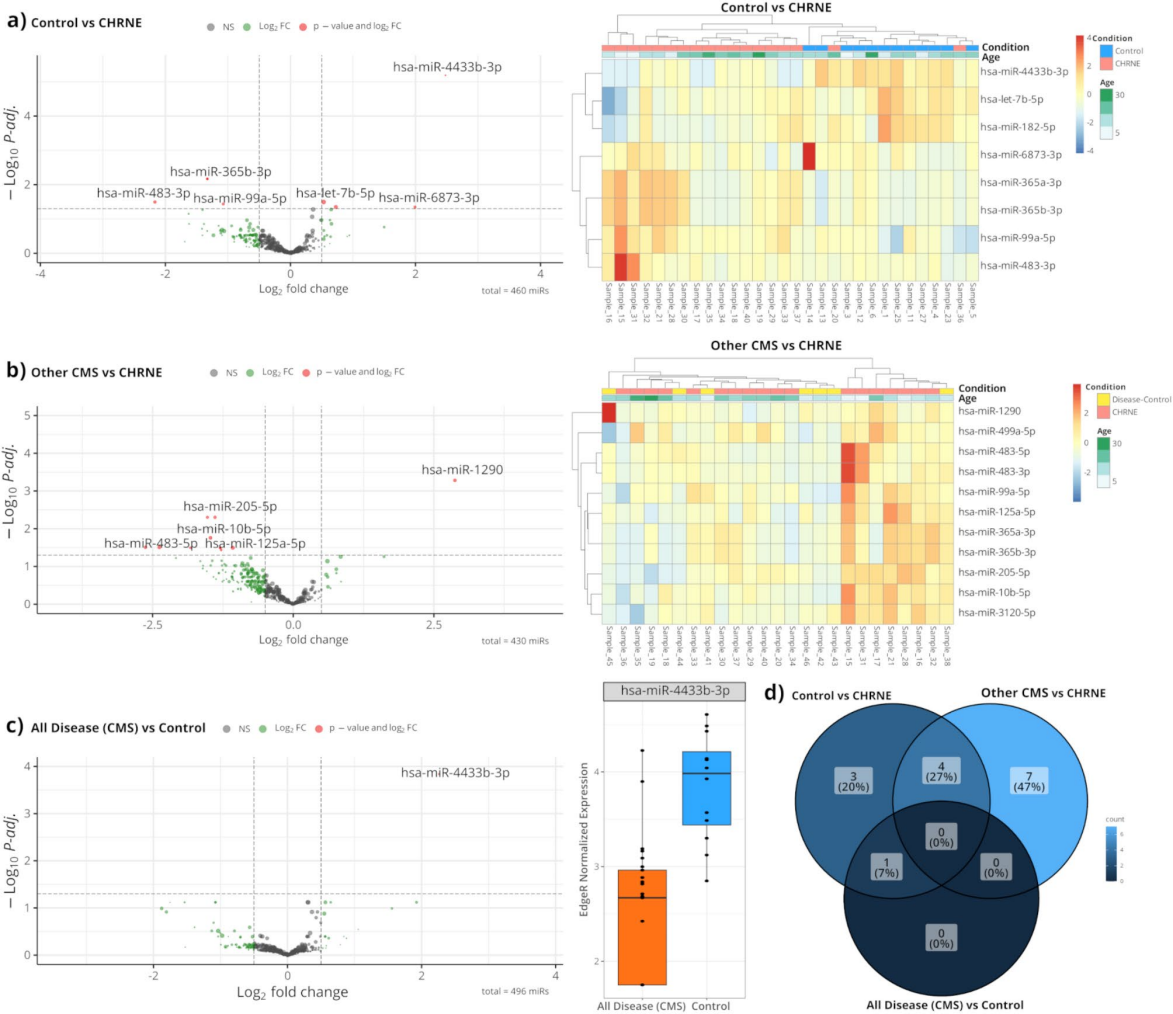
Differentially expressed miRNAs (DEmiRNAs) between *CHRNE*-patients, other CMS subtype patients and healthy controls. Volcanos and heatmaps of the top DEmiRNAs based on the adjusted *p value* (< 0.05) for (**a**) *CHRNE*-patients versus controls and (**b**) *CHRNE*-patients versus other CMS subtypes. In heatmaps, the *CHRNE* group is shown in red colour, the control group (healthy individuals) is shown in blue colour and the other CMS subtype group in yellow. The colour key panel shows the Z-score values calculated for each miRNA, by subtracting the row-mean and then dividing by the standard deviation. Z-scores describe the expression of each miRNA in relation to the mean. Overexpressed miRNAs are shown in red, underexpressed miRNAs in blue. White colour indicates expression change close to 0. Hierarchical clustering was performed for samples and miRNAs. (**c**) Comparison of *CHRNE*-patients and other CMS subtypes versus controls revealed only miR-4433b-3p to be differentially expressed. (**d**) Venn diagram showing the number of unique and common miRNAs that reached significace in the three comparisons, *CHRNE*-patients versus controls, *CHRNE*-patients versus other CMS subtypes, *CHRNE*-patients and other CMS subtypes versus controls

Given that serum miRNAs have been proven to serve as suitable biomarkers for several neurological diseases, we aimed to identify novel serum-based miRNA biomarkers for *CHRNE*-related CMS, through high-throughput small RNA sequencing not only including healthy but also disease controls (other CMS subtypes) (Fig. 5). Following bioinformatics analyses, we identified a signature of miRNAs that associate with *CHRNE*-patients compared to controls. More particularly, the levels of four miRNAs, miR-483-3p, miR-365a-3p, miR-365b-3p and miR-99a-5p were found to be increased in the serum of *CHRNE*-patients compared to healthy individuals (Fig. 5a). In contrast, miR-4433b-3p, miR-6873-3p, miR-182-5p and let-7b-5p levels were found to be decreased in *CHRNE*-patients compared to unaffected controls (Fig. 5a).

To investigate whether these eight miRNAs are *CHRNE*-CMS disease-characteristic, we next examined whether their levels are also altered when we compare *CHRNE*-patients with patients with other CMS subtypes. Comparison of *CHRNE*-patients versus patients with other CMS-subtypes showed that the four miRNAs, miR-483-3p, miR-365a-3p, miR-365b-3p and miR-99a-5p, identified to be elevated in *CHRNE*-patients compared to controls were also found to be elevated in *CHRNE*-patients when compared to patients with other CMS-subtypes (Fig. 5b). These results imply that these four miRNAs may serve as *CHRNE*-associated miRNA biomarkers. Along this line, the levels of miR-182-5p and let-7b-5p found to be decreased in *CHRNE*-patients compared to healthy individuals, were also found to be decreased in *CHRNE*-patients versus patients with other CMS-subtypes. The decrease and adjusted *p. value* were slightly below in the list with our selection criteria (|log2FC|=0.58 and adjusted *p.value* = 0.16 for miR-182-5p and |log2FC|=0.6 and adjusted *p. value* = 0.07 for let-7b-5p) suggesting that they need to be further examined in larger number of samples. The other two miRNAs found to be decreased in *CHRNE*-patients compared to healthy individuals, miR-4433b-3p and miR-6873-3p showed no change in their levels when we compared *CHRNE*-patients versus patients with other CMS-subtypes. Seven other additional miRNAs were found to be altered between the *CHRNE*-patients and the patients with other CMS subtypes: our results show that miR-10b-5p, miR-205-5p, miR-125a-5p, miR-483-5p, miR-499a-5p and miR-3120-5p levels were found to be elevated in the *CHRNE*-patients compared to patients with other CMS-subtypes (Fig. 5b). On the other hand, miR-1290 levels were found to be decreased in the *CHRNE*-patients compared to patients with other CMS-subtypes (Fig. 5b). The levels of four of these miRNAs, miR-10b-5p, miR-125a-5p, miR-483-5p and miR-499a-5p, were also found to be elevated in *CHRNE*-patients compared to healthy individuals (|log2FC| > 0.5) although they showed lower adjusted p-values. The other three miRNAs, miR-1290, miR-205-5p and miR-3120-5p, showed no significant differences between the *CHRNE*-patients and control groups.

We next examined whether there are miRNAs that can be used as biomarkers for all the CMS subtypes and thus grouped the *CHRNE*-patients with the patients with other CMS subtypes and we compared them with the healthy controls. This analysis revealed that miR-4433b-3p was decreased in all the patients compared to controls thus suggesting that this miRNA can serve as a CMS-associated miRNA biomarker (Fig. 5c).

**Fig. 6:**
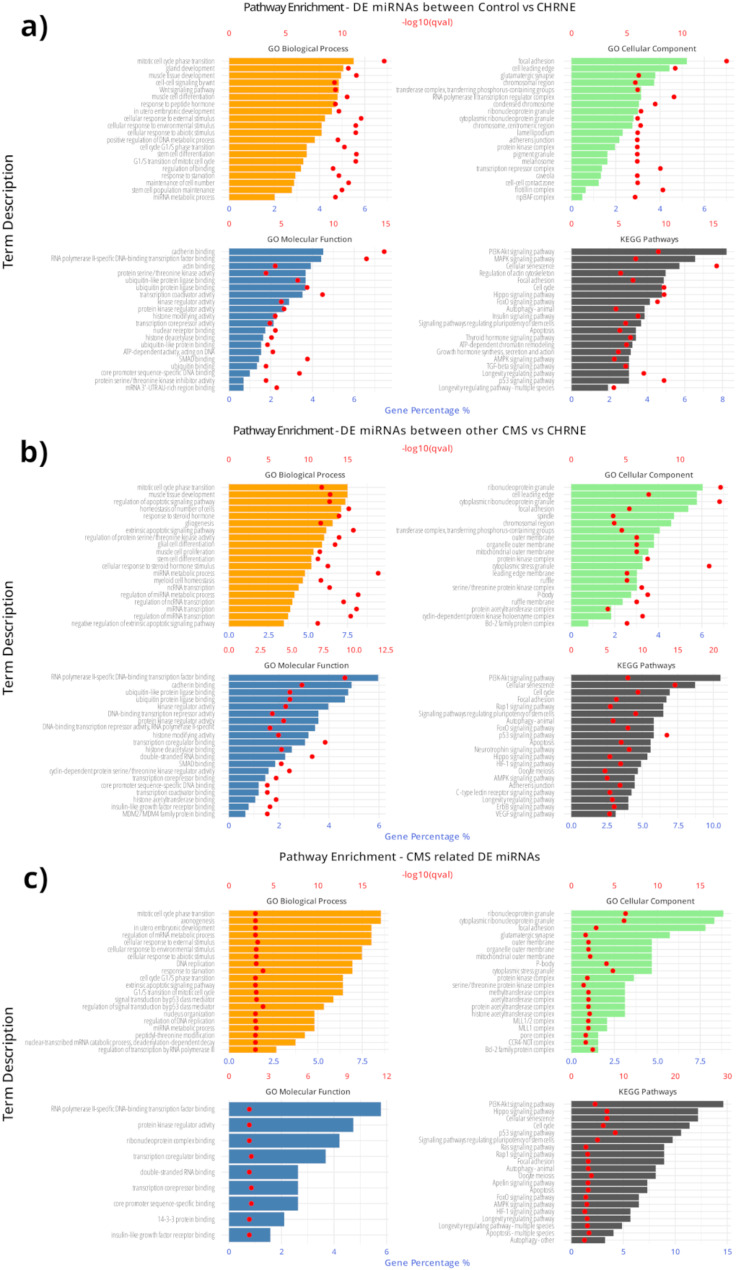
Enriched biological processes based on the experimentally validated target genes of the DEmiRNAs. Gene target of the DEmiRNAs and pathway analysis of the experimentally validated target genes of the differentially expressed miRNAs between (**a**) *CHRNE*-patients versus controls, (**b**) *CHRNE*- patents versus other CMS subtype patients. (**c**) Functional analysis of the experimentally validated target genes of the DEmiRNAs in both comparisons, *CHRNE*-patients versus controls and *CHRNE*-patients versus other CMS subtype patients. For the functional analysis, the top 20 enriched ontologies from GO biological processes, GO cellular components, GO molecular function and KEGG pathways are present

To investigate the potential functional and biological impact when these gene targets are dysregulated, an in silico based functional analysis through the GO knowledgebase and KEGG PATHWAY Database were next performed. To this end, functional analysis of the experimentally validated target genes of the miRNAs identified to be altered in *CHRNE*-patients compared to healthy individuals (Fig. 6a) was carried out in addition to an analysis focussing on targets identified by the comparison of *CHRNE*-patients and other CMS-subtypes (Fig. 6b) were performed. Furthermore, functional analysis of the experimentally validated target genes of the miRNAs (miR-483-3p, miR-365a-3p, miR-365b-3p and miR-99a-5p) found to possibly be *CHRNE*- specific based on the comparison of signatures between *CHRNE*-patients and combined healthy and disease controls, was performed showing that Apelin-signalling is a process only found in *CHRNE*-patients (Fig. 6c). Functional analysis of the experimentally validated target genes of miR-4433b-3p which may serve as a CMS-associated miRNA biomarker revealed no results. The top 20 enriched ontologies from GO biological processes, GO cellular components, GO molecular function and KEGG pathways were identified for each comparison. The full set of the gene targets and the corresponding functional analysis are given in supplementary Tables 5 to 7.

## Discussion

CMS represents a group of rare neuromuscular conditions caused by pathogenic variants affecting genes encoding for proteins playing crucial roles at the NMJ. Currently, more than 35 disease causative genes have been described whereby *CHRNE* seems to be the most prevalent form [25–27]. Although the underlying pathophysiology is well understood, there is currently still a lack of minimal-invasive biomarkers ideally allowing to also obtain insights into the molecular etiology of *CHRNE*-related CMS. To systematically address this lack of informative biomarker signatures, we conducted a comprehensive study applying different omics-technologies to decipher proteins, metabolites and miRNAs associated with disease manifestation in *CHRNE*-related CMS. A total of 19 patients (from 13 different families) with AChR deficiency due to biallelic, pathogenic *CHRNE* variants were included in the overall study. Twelve families harbour pathogenic variants (*n* = 7) already described in literature. One family presents with a novel homozygous variant (NM_000080.4:c.1032 + 2_1032 + 3delinsGT, r.spl?, p.?), expanding the current genetic landscape of *CHRNE*-related CMS. This variant segregates in a family including three affected individuals, two with a mild clinical phenotype (patients 4 & 6) and one severely affected one (patient 5). The latter patient, in contrast to his siblings, showed also intellectual disability. Phenotypes observed in our cohort varied from isolated ocular symptoms (ophthalmoparesis and fluctuating ptosis) to pronounced muscular weakness with reduced walking distance and wheelchair requirement (noted in 2 patients). In accordance with previous clinical observations reported in literature [8], we also observed a progressive muscular weakness in some patients, despite the ongoing therapy – although some cases were even treated with 3 different drugs (see Table 1). Also in accordance with previous clinical observations [4, 8, 10], familial phenotypic discordance has been noted in our cohort for two families. Therefore, additional genetic and/ or biochemical factors (in terms of modifiers) are presumed to be responsible for the variable familial phenotypes. Previously, a gender effect was postulated to be responsible for intrafamilar manifestation in siblings with pathogenic variants in either *MUSK* or *CHRNE*, whereby female patients were more severely affected than the males [28, 29]. Although in our *CHRNE* cohort of severely affected patients, 3 out of 4 were female, the overall number of patients is too small to draw final conclusions regarding a gender influence. Intellectual disability was observed in a total of four patients from consanguineous families. Given that *CHRNE* is not expressed in the central nervous system, we assume that this clinical feature mostly likely results from additional genetic defects in terms of genetic double-trouble or acquired causes. Moreover, genotypes resulting from consanguinity might also impact on the apparent severity of the neuromuscular phenotype (for example patient 5). However, exome sequencing was not performed in these patients to prove this assumption.

Interestingly, although one might assume that disease severity may potentially be related to response to standard therapeutic intervention utilizing Acetylcholinesterase (AChE) inhibitors, a clear statistically significant correlation between severity and pyridostigmine treatment was not observed in the context of a recent study [30]. Nonetheless, the same study unveiled that within the severe group of CMS-patients, significant enrichment in genes belonging to the extracellular matrix (ECM) pathway (in particular interactions of ECM receptors and ECM proteoglycans) was identified. This in turn might lead to disruptions in NMJ functionality [30]. Hence, these findings suggest the presence of a functional relevant network of protein dysregulations decisive for clinical manifestation and even disease severity. To decipher the impact of dysregulation of molecular determinants for disease manifestation in *CHRNE*-related CMS, in the framework of the study presented, omics technologies have been applied on blood samples. This approach also aimed toward the definition of minimal-invasive biomarker signatures which may even have a pathophysiological impact. We divided the *CHRNE*-patients of our overall cohort into two larger groups depending on their symptom severity and performed proteomic analyses of WBC and EVs in addition to metabolomic and miRNA transcriptomic studies on sera. Our proteomic profiling approach enabled the identification of 43 proteins with significantly altered abundances in WBC derived from *CHRNE*-patients impacting on diverse cellular processes including mitochondrial activity but also secretory granules and the extracellular space. To hereby decipher proteins of profound clinical relevance, we compared the proteomic signature between patients mildly versus severely affected in our overall cohort. This strategy unravelled increase of two vesicular transport proteins, SCAMP2 and SNX2, in the severely affected patients. A larger cohort of patients would be needed to confirm their potential to serve as stratification markers. Nevertheless, as this finding was indicative for affection of the vesicular transport machinery, we next investigated extracellular vesicles purified from sera samples derived form 7 *CHRNE*-patients of our cohort. No changes were identified in the physical characteristics of patient-derived vesicles compared to controls, but proteomic profiling showed a significant dysregulation of 20 EV-proteins in *CHRNE*-patients. Interestingly the proteins identified with increased abundance included TARSH, a secreted protein known to promote the reduction of mitral cell dendritic complexity and to restrict dendritic branching and outgrowth of interneurons [22]. Moreover, ATRN and PLEC were decreased. ATRN has a critical role in normal myelination in the central nervous system [21], and genetic variants affecting *Atrn* impair working memory [31] and disturb reactive-oxygen-species (ROS) metabolism in skeletal muscles [32] in rats. PLEC serves as an intermediate filament and NMJ integrity requires linkage of AChR to postsynaptic intermediate filament networks via PLEC [33]. Taking the different roles of these three proteins in neurological functions into consideration, we addressed the question whether their abundance my also correlate with intellectual disability in a sub-cohort of our patients. However, no differences were observed in the level of these three proteins between *CHRNE*-patients with cognitive impairment compared to such with normal intelligence. The small number of samples must hereby also be taken into account and studies on larger cohorts are required to draw further conclusions. In any case, our combined proteomic results show that minimal-invasive proteinogenic markers can be identified in the blood of *CHRNE*-patients and may even provide an indication of an alteration in vesicular transport as part of the underlying pathophysiology. Worth noting, that altered vesicular transport affecting agrin secretion has already been described in an in vitro model of *MYO9A*-related CMS [11]. Doubtless, further pathophysiological studies are needed to precisely decipher the impact of altered vesicle transport and protein release in the molecular etiology of *CHRNE*-related CMS.

Given that a recent publication highlighted on coupling of multilayer analysis with personalized omics information to provide molecular explanations to underlying pathophysiologies in CMS [30], in addition to proteomic studies, we performed metabolic profiling on serum samples derived from 9 *CHRNE*-patients. This omics-based approach enabled the identification of decreased level of seven amino acids/ amino acid intermediates hinting toward metabolic changes as a molecular fingerprint underlying in *CHRNE*-related CMS. Notably, one of the most important metabolic processes to provide energy in the cell is amino acid metabolism and almost all of the 20 amino acids that serve as the building blocks of proteins are produced or degraded in the mitochondria [34]. Along this line, our global proteomic profiling data on WBC highlight decrease of several mitochondrial proteins thus indicating a pathophysiological interplay between altered amino acid metabolism and mitochondrial maintenance and function. Among the dysregulated metabolites, 1-methlyhistitdine is decreased and altered 1-methlyhistitdine serum level were previously described in a mixed cohort (seropositive and seronegative) of 28 myasthenia gravis (MG) patients. However, in MG-patients, levels were slightly elevated [35]. From the pathophysiological point of view, decrease of phosphoserine is of interest: Wallace and colleagues recently reported on accumulation of pS129 at synapses in diseases like Morbus Parkinson and dementia with Lewy bodies with profound effects on vesicle dynamics [36] as a biological event also affected in blood of *CHRNE*-patients. In 2019, Simó and colleagues even highlighted that phosphorylation of serin-residues in SNAP-25 ensures an accurate transmission process at the NMJ [37] and one of our recent studies highlighted on the benefit of L-serin-treatment toward restoration of the NMJs in a zebrafish model of a rare neuropediatric disorder called Marinesco-Sjögren syndrome [38].

Given that thus far no miRNA biomarker has been described in the context of CMS, we applied a respective molecular study utilizing serum samples from *CHRNE*-patients in addition to healthy controls and biosamples derived from patients presenting with CMS based on other genotypes (serving as disease controls). Indeed, this approach enabled us to identify eight miRNAs differentiating between *CHRNE*-patients and healthy controls. Of note, six of these markers (upregulated: miR-483-3p, miR-365a-3p, miR-365b-3p and miR-99a-5p; downregulated: miR-182-5p and let-7b-5p) even maintain differentially abundant when comparing *CHRNE*-patients with patients suffering from other CMS-subtypes suggesting their potential to serve as stratification markers. Along this line, a global comparison between *CHRNE*-patients and other and the mixed cohort of other CMS-subtypes unveiled seven additional miRNAs to be different between these two groups (upregulation: miR-10b-5p, miR-205-5p, miR-125a-5p, miR-483-5p, miR-499a-5p and miR-3120-5p; downregulation: miR-1290). Hence, our approach introduced a set of miRNA as minimal-invasive biomarkers enabling a stratification of *CHRNE*-patients, an important aspect not only the potential molecular monitoring of therapeutic intervention concepts but also in the evaluation of *CHRNE*-variants of unknown significance. However, further studies are needed to investigate whether these miRNAs (each on its own or as a set) may enable to differentiate between the two main subtypes caused by *CHRNE*-variants, AchR deficiency patients based on bi-allelic variants (as introduced here) and slow-channel patients based on heterozygous variants of dominant nature. Regarding the potential impact on the underlying pathophysiology, it is important to note that miRNAs dysregulated between *CHRNE*-patients and healthy controls not only impact on cell cycle and different signalling pathways (i.e. AMPK-, PI3K-AKT-, MAPK-, Hippo- and FoxO-signalling), but also on muscle cell development and function of glutamatergic synapses among others. These signalling cascades were already described as mechanisms regulating NMJ development and function and causing muscle wasting (for overview: [39, 40]). According to the results of our KEGG pathway analysis, miRNAs enabling a differentiation between *CHRNE*- and other CMS-patients target (in addition) certain other signalling cascades including HIF1-, Rap1- and ErbB-signalling in addition to neurotrophin signalling. This may hint toward the presence of partially overlapping but also slightly diverging signalling cascades affected by the different subtypes of CMS in terms of molecular drivers of NMJ-vulnerability and hence clinical manifestation. Doubtless, further functional studies (on animal models; [41]) are crucial to molecularly dissect the impact of these different signalling cascades in the etiology of the different subtypes and thus to define a molecular map ideally linking both, miRNA dysregulations and the respective impact on the target proteins modulating these signalling cascades. Our miRNA discovery approach additionally identified miR-4433b-3p, which targets CNDP2 [42], as a miRNA generally decreased in CMS-patients irrespective of the underlying genotype. Given that CNDP2 was linked to the production of Lac-Phe (from lactate and phenylalanine), a molecular effector associated with physical activity [43], general decrease observed across the overall CMS-cohort might reflect restraints in physical activities. Accordingly, it would be interesting to study the level in CMS-patients therapy-naïve and under therapy in correlation with clinical outcomes and benefits to address the potential of miR-4433b-3p to serve as a generalized and minimal-invasive therapy biomarker. Moreover, this would enable to examine its potential to serve as a valid myomiR, in terms of a muscle-enriched microRNA acting as an effective regulator of muscle homeostasis in both physiological and pathological conditions. Interestingly, in *CHRNE*-patients, four miRNAs (miR-483-3p, miR-365a-3p, miR-365b-3p and miR-99a-5p) distinguish from both, healthy and disease controls hinting toward a *CHRNE*-characteristic molecular signature which is based on the results of our in silico driven studies impacting on Apelin signalling which is not affected in the other comparisons. Apelin regulates skeletal muscle adaptation to exercise [44] and Apelin resistance contributes to cachexia-induced muscle loss [45].

### Limitations

Our study has several limitations related to the study design with a one-time-point evaluation. Not all patients included in all sub-studies based on limited biomaterial were available. Different aspects such as age, fold and duration of improper muscle innervation, physical activity levels and muscle mass might represent significant confounding factors. Longitudinal sample collection over longer period of time in correlation with clinical symptoms would be crucial to address the potential of these biochemical markers to even serve as progression markers. Moreover, further functional studies are needed to decipher the role of CHRNE in white blood cell populations and thus to fully interpret our identified biochemical dysregulations in a robust pathophysiological context. Along this line, abundance and distribution studies of WBC dysregulated proteins with impact on vesicle transport, myelination, synaptogenesis, and synaptic function in CMS models (such as *Chrne*-deficient mice) are needed to demonstrate their pathophysiological impact in the molecular etiology of CHRNE-related CMS.

On a general note, it should be considered that rare diseases are especially challenging to establish biomarkers based on the frequency of the respective diseases in combination to the availability of biomaterial needed for robust biomarker research. This in fact is certainly a general limitation for the neuromuscular field. Hence, based on the limitation of biomaterial, our study lacks a control cohort to validate the omics findings by making use of alternative analytical approaches.

## Conclusions

Our combined studies – for the first time – introduce a set of minimal-invasive biomarkers of potential pathophysiological relevance in *CHRNE*-related CMS across different molecular layers including proteins, metabolites and miRNAs. Hereby, results of our global proteomic profiling approach hinted toward altered vesicular transport in WBC derived from *CHRNE*-patients and study of the proteomic signature of EVs purified from sera derived from these patients indeed confirmed altered abundances of different proteins including such with known roles for NMJ-integrity and -function. Results of our metabolic studies unveiled metabolic changes in *CHRNE*-patients whereby some of these changes (such as serin-homeostasis) may also have pathophysiological impacts. Our miRNA profiling approach led to the identification of a set of miRNAs not only enabling a distinguishment between *CHRNE*-patients and healthy controls but also between these patients and patients suffering from other subtypes of CMS. Further comparison unveiled for miRNAs (miR-483-3p, miR-365a-3p, miR-365b-3p and miR-99a-5p) only being dysregulated in *CHRNE*-patients. Also, here targets of these miRNAs might contribute to the pathophysiology of CMS related to bi-allelic defects in *CHRNE* and other genes. Hence, our combined data introduce a catalogue of blood biomarkers which (as a set or on their own) may enable new perspectives in diagnosis, monitoring and stratification of *CHRNE*-related CMS patients.

## Electronic supplementary material

Below is the link to the electronic supplementary material.

Supplementary Material 1: Table 1: Demographic and clinical data of CMS disease controls included in the study.

Supplementary Material 2: Table 2: List of proteins identified as statistically significant dysregulated in white blood cells derived from*CHRNE*-patients by unbiased proteomic profiling.

Supplementary Material 3: table 3: list of proteins identified as statistically significant dysregulated in EVs purified from sera of CHRNE-patients by unbiased proteomic profiling.

Supplementary Material 4: Table 4: List of metabolites identified as statistically significant dysregulated in sera of*CHRNE*-patients by unbiased mass spectrometry.

Supplementary Material 5: Table 5: Full set of the gene targets and the corresponding functional analysis for the comparioson of miRNA signatures in blood of *CHRNE*-patients versus healthy controls.

Supplementary Material 6: Table 6: Full set of the gene targets and the corresponding functional analysis for the comparioson of miRNA signatures in blood of*CHRNE*-patients versus further CMS patients servin as disease controls.

Supplementary Material 7: Table 7: Full set of the gene targets and the corresponding functional analysis for the comparioson of miRNA signatures in blood of all CMS patients versus healthy controls.

Supplementary Material 8: Fig. 1: Schematic overview of biosamples included for the different applied analytical approaches involving such derived from *CHRNE*-patients, normal disease controls (NDC) and additional CMS genotypes serving as diseases controls (CMS disease controls = CMS-DC).

## Data Availability

Data is provided within the supplementary information files.

## Abbreviations

AChE: Acetylcholinesterase
AChR: Acetyl-Choline-Receptor
ACMG: American College of Medical Genetics and Genomics
AMP: Association for Molecular Pathology
ATRN: Attractin
CC: Cellular component
CMS: Congenital myasthenic syndrome
DAP: Diaminopyridine
DDA: Data-dependent acquisition
DE: Differential expressional
DIA: Data-independent acquisition
ECM: Extracellular matrix
EVs: Extracellular vesicles
FDR: False discovery rate
GO: Gene Ontology
log2FC: log2 Fold Change
MF: Molecular function
MG: Myasthenia gravis
NMJ: Neuromuscular junction
ORA: Over Representation analysis
PASEF: Parallel accumulation–serial fragmentation
PBS: Phosphate buffered saline
PLEC: Plectin
PSM: Peptide-spectrum matches
PS: Pyridostigmine
ROS: Reactive-oxygen-species
SEC: Size exclusion chromatography
TARSH: Target of Nesh-SH3
WBC: White blood cells
