# Supplementary material for: Blood biomarker fingerprints in a cohort of patients with CHRNE-related congenital myasthenic syndrome
Source: Acta Neuropathol Commun. 2025 Feb 13;13:29. doi: 10.1186/s40478-025-01946-9 (PMC11823195; doi:10.1186/s40478-025-01946-9)
Supplement: Supplementary file 1 — Supplementary Material 1: Table 1: Demographic and clinical data of CMS disease controls included in the study. [file 40478_2025_1946_MOESM1_ESM.docx]

Supplementary Table 1

| *Disease control* | *Genetic variant* | *Sex* | *Age* | *Age symptom onset* | *Reduced walking distance* | *Wheelchair* | *Therapy* | *Severity* | *Analytical approaches* |
| --- | --- | --- | --- | --- | --- | --- | --- | --- | --- |
| 1D | *CHRNB1* | m | 3 | infant | + | - | PS, Sal | 2 | miRNA, EV |
| 2D | *GFPT1* | m | 5 | infant | + | - | PS | 2 | miRNA |
| 3D | *CHAT* | m | 9 | 0 | ++ | + | PS | 3 | miRNA, EV |
| 4D | *GFPT1* | m | 10 | 2 | +++ | + | PS | 3 | miRNA |
| 5D | *CHRNA1* | f | 13 | 5 | + | - | PS, Eph | 2 | miRNA |
| 6D | *SLC18A3* | m | 13 | 0 | no walking | + | PS | 4 | miRNA, EV |
| 7D | *CHRNE*slowch | m | 7 | infant | + | - | none | 2 | miRNA |
| 8D | CHAT | m | 17 | infant | no walking | + | PS, 3,4 DAP | 4 | EV |
