# Supplementary figures and images for: Blood biomarker fingerprints in a cohort of patients with CHRNE-related congenital myasthenic syndrome
Source: Acta Neuropathol Commun. 2025 Feb 13;13:29. doi: 10.1186/s40478-025-01946-9 (PMC11823195; doi:10.1186/s40478-025-01946-9)

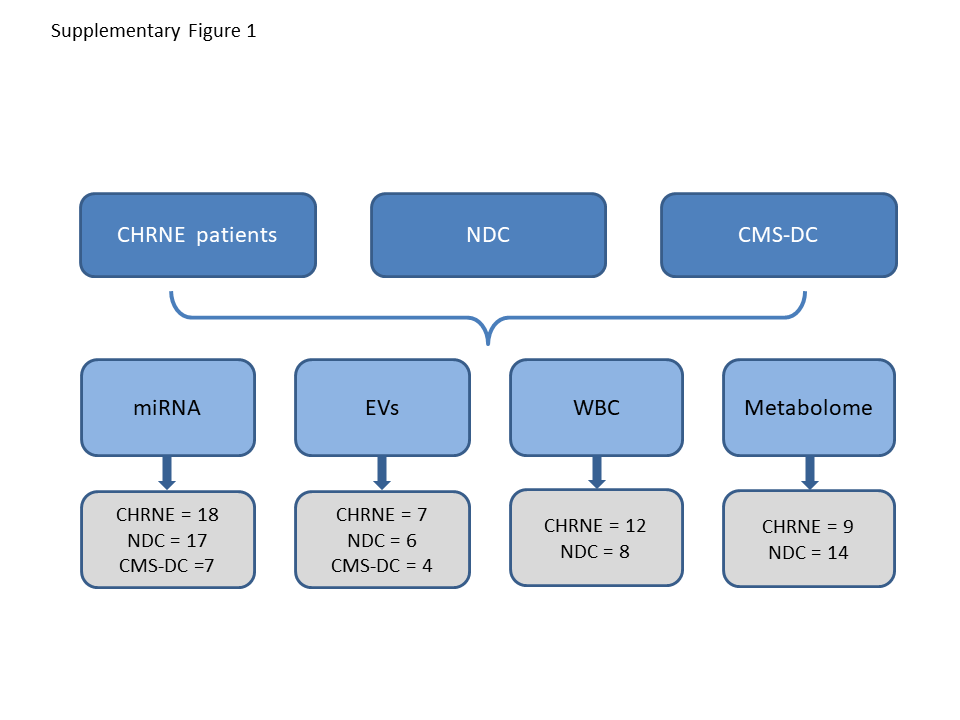

Supplement: Supplementary file 8 — Supplementary Material 8: Fig. 1: Schematic overview of biosamples included for the different applied analytical approaches involving such derived from CHRNE-patients, normal disease controls (NDC) and additional CMS genotypes serving as diseases controls (CMS disease controls = CMS-DC). [file 40478_2025_1946_MOESM8_ESM.tif]
